# Development of Optical-Based Molecularly Imprinted Nanosensors for Adenosine Detection

**DOI:** 10.1021/acsomega.3c01028

**Published:** 2023-05-18

**Authors:** Zehra Tuğçe Kurt, Duygu Çimen, Adil Denizli, Nilay Bereli

**Affiliations:** † Bioengineering Division, Hacettepe University, Ankara 06230, Turkey; ‡ Department of Chemistry, Hacettepe University, Ankara 06800, Turkey

## Abstract

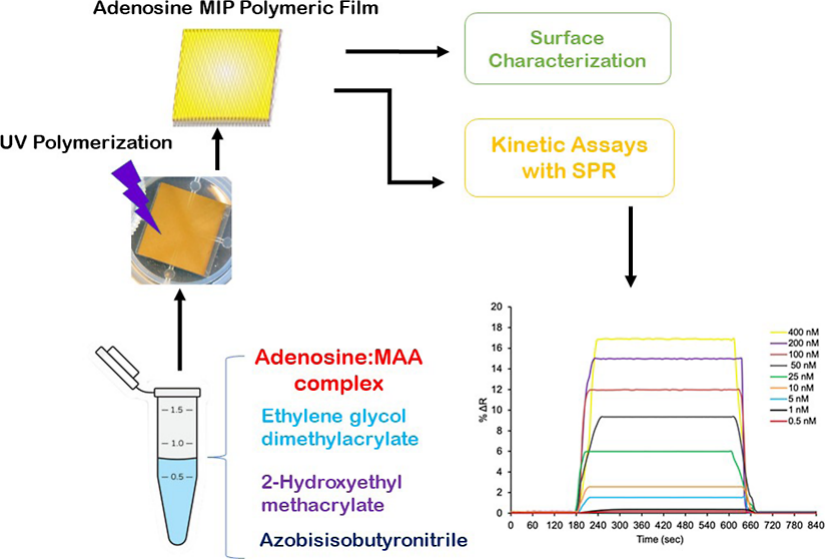

Adenosine nucleoside is an important molecule in human physiology. The levels of adenosine nucleoside in urine and plasma are directly or indirectly related to diseases such as neurodegenerative diseases and cancer. In the present study, adenosine-imprinted and non-imprinted poly(2-hydroxyethyl methacrylate-methacrylic acid) (poly(HEMA-MAA)) surface plasmon resonance (SPR) nanosensors were prepared for the determination of adenosine nucleoside. First, MAA/adenosine pre-polymerization complexes were prepared at different molar ratios using adenosine as a template molecule and methacrylic acid (MAA) as a monomer, and SPR nanosensor surfaces were optimized by determining the highest imprinting factor of the chip surfaces. The surfaces of adenosine-imprinted and non-imprinted SPR nanosensors were characterized by using atomic force microscopy, ellipsometry, and contact angle measurements. Kinetic analyses were made with different concentrations in the range of 0.5–400.0 nM for the detection range with a pH 7.4 phosphate buffer solution. The limit of detection in adenosine aqueous solutions, artificial plasma, and artificial urine was determined to be 0.018, 0.015, and 0.013 nM, respectively. In the selectivity analysis of the developed nanosensors, the selectivity of adenosine SPR nanosensors in solutions at different concentrations was determined by using guanosine and cytidine nucleosides. The relative selectivity coefficients of adenosine-imprinted SPR nanosensors for adenosine/cytidine and adenosine/guanosine are 3.836 and 3.427, respectively. Since adenosine-imprinted SPR nanosensors are intended to be used in medical analysis and research, adenosine analysis has also been studied in artificial urine and artificial plasma samples.

## Introduction

Adenosine (Ado) nucleoside is an important endogenous purine nucleoside consisting of an adenine molecule attached to a ribose sugar moiety via a beta-N9-glycosidic bond. Adenosine, which has main functions in human physiology, is also an extracellular signaling molecule.^1^ It is found in all tissues of vertebrates, including the central nervous system, and regulates various physiological processes.^2^ It is directly involved in a number of functions, including metabolism, cellular communication, and DNA methylation.^3^ It has been emphasized that adenosine plays an important role in diabetes, autoimmune diseases, cardiovascular disease, as well as neurological diseases such as epilepsy and Alzheimer’s anxiety and many cancer types such as colorectal cancer, breast cancer, prostate cancer, and lung cancer.^4−19^ It has been reported that the concentration of adenosine in plasma and urine increases under some conditions related to diseases. Thus, adenosine has a biomarker feature^20^ according to urine and plasma levels. Due to the half-life of adenosine, the concentration range in plasma is generally 10 nmol/L to 1 μmol/L. The in vitro plasma levels of adenosine were found to be 82 ± 14 nM. In general, the concentration of adenosine in normal human urine ranges from 2.0 to 7.0 μM, and the reference limit of adenosine in a lung cancer patient is higher than 7.0 μM.^21^ It has been observed that the adenosine concentration in urine increases in type 1 diabetes, patients due to diabetes.^22^ Besides, it was stated that the level of adenosine concentration increased in patients with heart failure and lung failure.^23−25^ Methods such as liquid chromatography tandem mass spectrometry (LC–MS/MS), high-performance liquid chromatography (HPLC), and matrix-assisted laser desorption/ionization time-of-flight (MALDI-TOF) are used for analytical determination of adenosine.^26−28^ However, the surface plasmon resonance (SPR) nanosensor is a real-time, fast, sensitive, and low-cost method compared to other analytical methods.^29−31^ Another reason why the SPR nanosensor is advantageous and effective is that it is widely used in studies with molecularly imprinted polymers (MIPs).^32,33^

SPR nanosensors measure changes in refractive index that occur on the surface of a metal film on which electromagnetic waves, called surface plasmons, are propagated.^34^ The SPR detection stage generally allows the light from the light source to pass through the glass prism, which has a high refractive index, to stimulate the surface plasmon with the angle of incidence. After the interaction of the incident light wave with the special recognition regions on the gold film surface, it causes a decrease in the reflected light intensity, and the reflected light is detected in the detector.^32^ By enabling real-time investigation of biomolecular interactions, SPR nanosensors have become an important tool in medical diagnosis.^35^ The SPR system, which provides real-time results in research and disease diagnosis, can easily diagnose and detect molecules in nanostructures. In recent years, the use of molecular imprinting technology together with the SPR system provides real-time and effective results in analytical solutions.

Molecular imprinting technology (MIT) for biomarkers is very advantageous because it creates highly selective regions and is simple, fast, and convenient.^36,37^ The polymeric structure is formed as a result of polymerization around a functional monomer and a cross-linked template molecule by molecular imprinting technology. Three-dimensional selective recognition regions are formed from the size, shape, and functional groups created specifically for the target regions.^38^ MIPs are synthetic materials that have artificial recognition sites by specifically rebinding a target compound or a molecule.^39^ In general, the preparation process of MIPs is carried out by adding the pre-complex between the functional monomer and the template, the cross-linking agent and initiators, and the selective sites formed on the surface to be washed away from the non-interacting substances.^40^

This study aimed to detect adenosine nucleoside in real time. For this purpose, adenosine was imprinted by the molecular imprinting method on the gold chip surface activated with allyl mercaptan. The adenosine-imprinted poly(2-hydroxyethyl methacrylate-methacrylic acid) (poly(HEMA-MAA)) polymeric film was prepared for adenosine detection in artificial plasma and artificial urine. Adenosine-imprinted and non-imprinted SPR nanosensors, which were modified and made ready as a result of characterization studies such as atomic force microscopy (AFM), ellipsometry, and contact angle measurements, were analyzed with a SPR device for real-time detection of adenosine. After determining the optimal pH, kinetic analyses were carried out in the concentration range of 0.5–400 nM. Guanosine and cytidine nucleosides were used as competitor agents to determine the selectivity of the adenosine-imprinted SPR nanosensor. In addition, the shelf life and reusability studies of SPR nanosensors were also carried out. The applicability of the adenosine-imprinted SPR nanosensor in the real environment, the limit of detection (LOD), and quantification (LOQ) were determined by the adenosine amount in artificial urine and artificial plasma.

## Materials and Methods

### Chemicals

Adenosine, methacrylic acid (MAA), ethylene glycol dimethacrylate (EGDMA), 2,2′-azobisizobutyronitrile (AIBN), 2-hydroxyethylmethacrylate (HEMA), guanosine and cytidine nucleosides for selectivity analysis, acetate buffer, phosphate buffer, ammonium chloride (NH_4_Cl), dipotassium phosphate (K_2_HPO_4_), monopotassium phosphate (KH_2_PO_4_), sodium sulfate (Na_2_SO_4_), magnesium sulfate (MgSO_4_), calcium chloride (CaCl_2_), urea (CH_4_N_2_O), sodium bicarbonate (NaHCO_3_), citric acid (C_6_H_8_O_7_), allyl mercaptan, and artificial plasma were obtained from Sigma (Chemical Co., USA). All chemicals are of analytical grade purity. The water purification processes high-flow cellulose acetate membrane (Barnstead D2731), reverse osmosis Barnstead (Dubuque, IA) Ropure LP unit, Barnstead D2731D3804 NANOpure organic/colloid removal unit, and ion-exchange packed column system were used during the experiments.

### Instruments

Bare gold SPR chip and SPR system were obtained from GenOptics, SPRiLab (Orsay, France). A spin coater device was used to distribute the adenosine-imprinted and non-imprinted polymer mixtures homogeneously on the SPR chip surface (Spin Coater, LAURELL, WS 650Mz-23NPP, USA). Characterization of SPR chip surfaces was performed with AFM (Nanomagnetics Instruments, Oxford, UK). The polymer thicknesses on adenosine-imprinted and non-imprinted SPR nanosensor surfaces was measured on an EP3-Nulling Ellipsometer (Göttingen, Germany). For the characterization of the bare gold SPR chip surface, adenosine-imprinted and non-imprinted SPR nanosensor surfaces were calculated with the Kruss DSA100 (Hamburg, Germany) contact angle device. After preparing and characterizing the adenosine-imprinted SPR nanosensor, kinetic studies were carried out with the GenOptics SPRiLab system.

### Activation of the SPR Gold Chip Surface

The SPR gold chip surface enables the chip surface to be activated to modification via allyl mercaptan (CH_2_CHCH_2_SH). The SPR gold chip surface was cleaned with acidic piranha solution, then washed with pure ethyl alcohol, and dried in an oven at 40 °C for 1 h. Allyl mercaptan was dripped onto the surface of the SPR gold chip, and it was kept for 1 day. Finally, the surface of the SPR gold chip modified with thiol (−SH) groups was cleaned with ethyl alcohol again to remove unbound allyl groups. Thus, the SPR chip surface is made ready for modification.

### Pre-polymerization Complex Formation with a Functional Monomer and a Template Molecule

While forming the MAA/Ado pre-polymerization complex with adenosine and methacrylic acid (MAA), MAA/Ado complexes with different molar ratios were formed to determine the stoichiometric coupling ratios. In order to determine the appropriate polymerization mixture, an adenosine-imprinted polymeric film was prepared in MAA/Ado molar ratios (1:1, 5:1, 10:1, and 20:1 M). SPR nanosensors designed with MAA/Ado (1:1, 5:1, 10:1, and 20:1 M) pre-polymerization complexes prepared in different molar ratios are named as MIP-1, MIP-2, MIP-3, and MIP-4 codes, respectively. A non-imprinted SPR nanosensor (NIP) was formed without the addition of adenosine by the same procedure. According to the results of kinetic analysis, since the highest signal can be seen at the ratio of 10:1 (MAA/Ado), imprinting factors were calculated with this ratio. Kinetic analyses were performed using a 10 nM adenosine aqueous solution to calculate the imprinting factor (IF: %Δ*R*_MIP_/%Δ*R*_NIP_).

### Preparation of Adenosine-Imprinted and Non-imprinted SPR Nanosensor Surfaces

First, the MAA monomer and adenosine at a ratio of 10:1 M were mixed in a glass vial to prepare the pre-polymerization complex. The pre-polymerization complex was mixed with 2-hydroxyethylmethacrylate (HEMA) as a functional monomer and ethylene glycol dimethacrylate (EGDMA) as a cross-linker, and the AIBN initiator was added to the final mixture.

**Figure 1:**
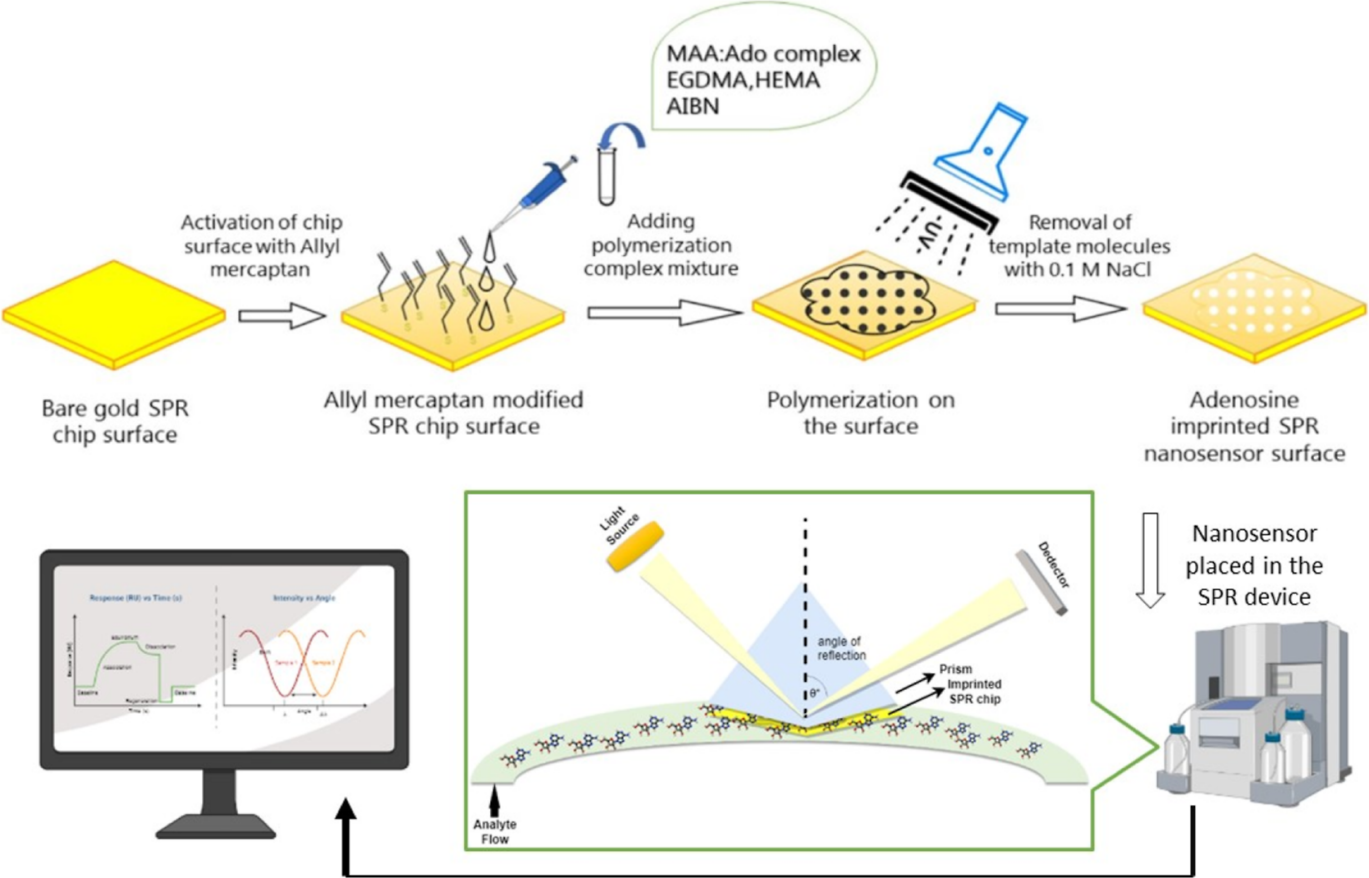
Schematic presentation of adenosine-imprinted SPR nanosensor surface preparation and kinetic analysis.

The prepared polymer mixture was dropped onto the ally mercaptan-modified SPR chip surface and homogeneously distributed on the SPR chip surface with a spin coater (Spin Coater, LAURELL, WS 650Mz-23NPP, USA). The polymerization mixture was immobilized on the SPR chip surface with a UV light for 30 min by the photopolymerization method. The adenosine-imprinted poly(2-hydroxyethyl methacrylate-methacrylic acid) (poly(HEMA-MAA)) SPR nanosensor (MIP) was shaken with a desorption solution at 200 rpm in a shaking incubator for 2 h. After the desorbed SPR nanosensor was washed with deionized water, it was dried in a vacuum oven (200 mmHg, 25 °C). A non-imprinted SPR nanosensor (NIP) was also prepared using the same polymerization procedure as MIP SPR nanosensor without using the template molecule adenosine (Figure 1).

### Characterization Studies of SPR Nanosensor Surfaces

Characterization of SPR nanosensor surfaces was performed using AFM (Nanomagnetics Instruments, Oxford, UK) in the semi-contact mode. The oscillation resonance frequency (341.30 kHz) was applied to the adenosine-imprinted and non-imprinted SPR sensor chips. The vibration amplitude is 1 VRMS, and the null vibration amplitude is 2 VRMS, and the samples were taken as images of 1 × 1 μm^2^ area at a scan rate of 2 μm/s and a resolution of 256 × 256 pixels.^41^

For the characterization of the ally mercaptan-modified SPR chip surface, adenosine-imprinted and non-imprinted SPR nanosensor surfaces were calculated with the Kruss DSA100 (Hamburg, Germany) contact angle device. A separate contact angle was determined for each image taken with the sessile drop method. The average values of the contact angles were obtained by taking the average of the two regions taken as the contact angle values, the left and right contact points. The contact angle values were calculated by averaging the 10 measurements taken for each.^42^

The surface thicknesses on the adenosine-imprinted and non-imprinted SPR nanosensors were measured on the EP3-Nulling Ellipsometer (Göttingen, Germany). Measurements were made at a wavelength of 532 nm and an incidence angle of 62° and repeated three times at six different points of SPR nanosensor surfaces. Results are reported as an average of the values received.^43^

### Kinetic Studies

The adenosine-imprinted SPR nanosensor was prepared, and the kinetic studies were carried out after characterization studies. At this stage, kinetic analyses of adenosine solutions prepared at different concentrations were performed with the SPR device at room temperature. During the study, the flow rate was 150 μL/min (0.031″ ID tubing); the operating wavelength was 800 nm, and the prism material was SF (silicone free) 10 glass (refractive index, RI = 1.720).^44^ The different acetate and phosphate buffers were prepared to find the appropriate pH range for kinetic analyses. Kinetic analyses were carried out by preparing an adenosine solution at a concentration of 10 nM using 0.1 M acetate buffer (pH 3.0, pH 4.0, and pH 5.0) and 0.1 M phosphate buffers (pH 6.0 and pH 7.4). Kinetic analyses were performed with the prepared adenosine solution in each different buffer solution. The changes in the refractive index (%Δ*R*) were observed with SPR sensorgrams throughout the kinetic analysis.

Before starting the kinetic analysis, the SPR nanosensor surface was passed through deionized water (10 mL). Then, the plasmon curve and refractive index changes (%Δ*R*) were taken, while the pH 7.4 phosphate buffer was passing through the system. By opening the kinetic imaging program of the SPR view software, pH 7.4 phosphate buffer was started to be given from the SPR system as equilibration buffer, and the equilibration process was continued for 3 min. After this step, adenosine solutions were prepared in the concentration range of 0.5–400 nM with pH 7.4 phosphate buffer and given to the SPR system for 8 min. The shift values occurring in the resonance frequency were monitored instantaneously, and when the equilibrium state was reached, the 0.1 M NaCl solution was passed through the system for 3 min to reach the desorption stage. Equilibration buffer and desorption solutions were passed through the system separately in each concentration analysis. The refractive index changes (%Δ*R*) were monitored by observing the SPR sensorgrams.

### Detection of Adenosine in Artificial Plasma and Artificial Urine

Artificial plasma is supplied ready for analytical analysis. The procedure for the preparation of artificial urine is dissolving 250 mL of water in 2.5 mM CaCl_2_, 2.0 mM citric acid, 170 mM urea, 25 mM NaHCO_3_, 90 mM NaCl, 2.0 mM MgSO_4_, 10 mM Na_2_SO_4_, 7 mM KH_2_PO_4_, 7 mM K_2_HPO_4_, and 25 mM NH_4_Cl. Its pH was adjusted to 6.0.^45^ 1 and 5 nM concentrations of the adenosine solution prepared in artificial plasma and artificial urine were added. First, the pH 7.4 phosphate buffer was passed through the SPR system for 3 min, and then the prepared adenosine-spiked plasma and urine solutions were passed through the SPR system for 8 min. In the last stage, the 0.1 M NaCl solution as a desorption agent was passed into the SPR system for 3 min. In the kinetic analysis, the refractive index changes (%Δ*R*) were monitored against time in the SPR device in real time at each step.

### Selectivity Tests

To determine the selectivity of the adenosine nanosensor, adenosine-imprinted and non-imprinted nanosensors were prepared. Kinetic analyses were performed using adenosine (Ado, MW: 267.24 g/mol), guanosine (Guo, MW: 283.241 g/mol), and cytidine (Cyd, MW: 243.217 g/mol) nucleosides. Guanosine and cytidine were chosen as competition molecules in the selectivity study because they are nucleosides similar to the adenosine molecule both in structure and molecular weight. The levels of the modified and non-modified structures of these three nucleosides are very important in both plasma and urine.^46−48^ First, adenosine, guanosine, and cytidine solutions at 50 nM concentration were passed through the SPR system separately. Afterward, the Cyd + Guo mixture and Ado + Cyd + Guo mixture at 50 nM concentration were passed through the SPR system, and SPR sensorgrams were monitored simultaneously.

### Reusability

To examine the reusability and shelf life of the adenosine-imprinted SPR nanosensor, kinetic analyses were performed on the same day and at different times using the same chip. In the reusability study, the adenosine solution was prepared at a concentration of 50 nM in a pH 7.4 phosphate buffer. First, the pH 7.4 phosphate solution was passed through the SPR system for 3 min to bring the system to equilibrium. After the SPR system reached equilibrium, the 50 nM adenosine solution was passed for 8 min. It was then passed with 0.1 M NaCl solution as a desorption solution for 3 min. The kinetic analysis is repeated four times in a row on the same day using the same chip. Moreover, adenosine aqueous solutions were prepared at a concentration of 50 nM at different times such as the first day, first month, second month, fourth month, and sixth month, and kinetic analyses were performed. The results of kinetic analysis were converted to SPR sensorgram %Δ*R* values. All steps were monitored in the SPR system in real time.

## Results and Discussion

### Characterization Studies

**Figure 2:**
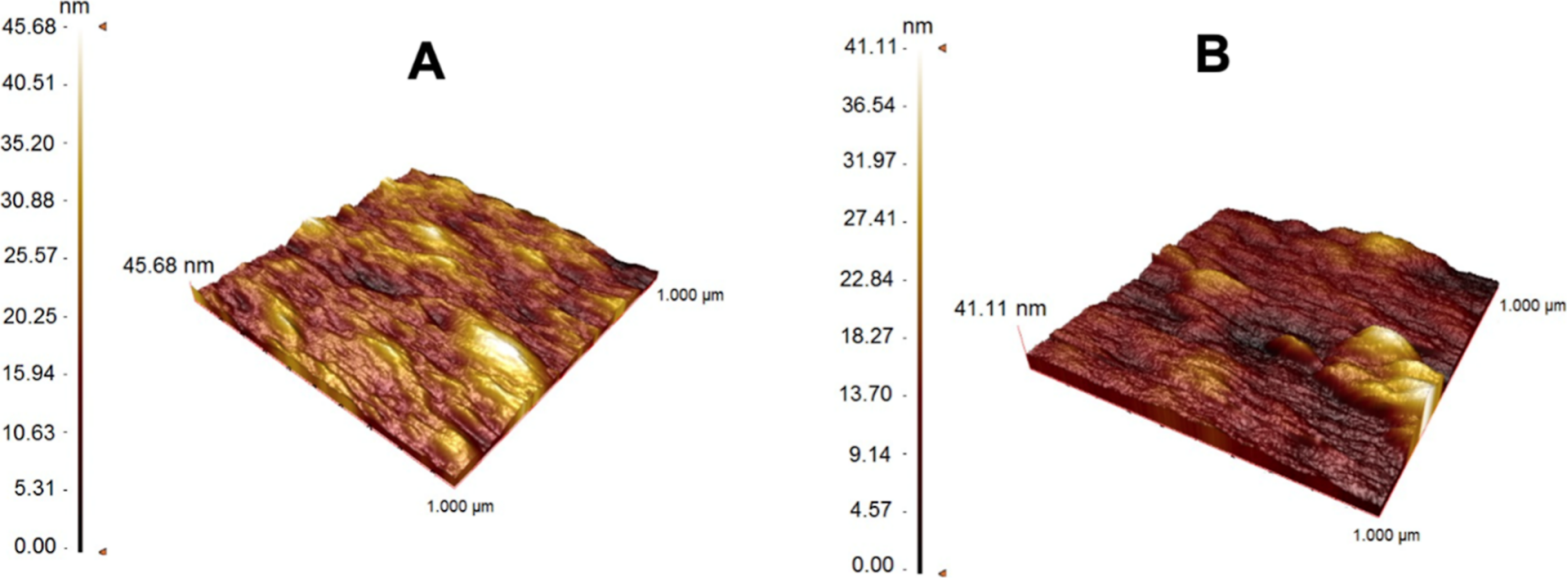
AFM image of adenosine-imprinted (A) and non-imprinted (B) SPR nanosensor surfaces.

The surface morphology of adenosine-imprinted and non-imprinted SPR nanosensors was characterized by AFM in the half-contact mode. According to the AFM imaging results, the surface roughness of adenosine-imprinted and non-imprinted SPR nanosensors was determined to be 45.68 and 41.11 nm, respectively (Figure 2). As can be seen in the AFM images in Figure 2, it can be seen that the polymeric film was synthesized homogeneously on adenosine-imprinted and non-imprinted SPR nanosensor surfaces.

**Figure 3:**
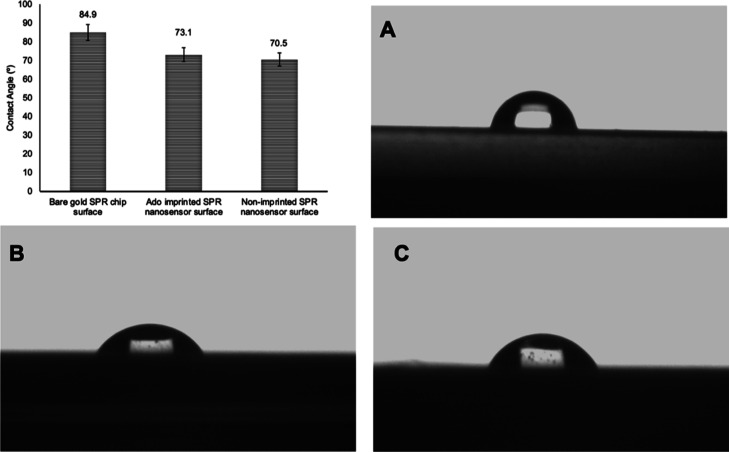
Surface contact angle image of the bare gold SPR chip surface (A), adenosine-imprinted (B), and non-imprinted SPR (C) nanosensor surfaces.

The wettability of the SPR nanosensor surface was determined by taking contact angle measurements with the sessile drop method. It is generally accepted that a surface is hydrophobic when the static water contact angle θ > 90° and is hydrophilic when θ < 90°.^49^ Characterization of SPR nanosensor surfaces was done by taking the average values of 10 measurements taken for each with the Kruss DSA100 (Hamburg, Germany) contact angle device (Figure 3). The contact angle of the bare SPR gold chip surface was 84.9°, while the contact angle of the adenosine-imprinted SPR nanosensor surface decreased to 73.1°. The contact angle value of the non-imprinted SPR nanosensor surface was determined to be 70.5°. As modifications are made on the SPR chip surface, it can be seen that the surface acquires a hydrophilic feature. The reason for the change in the SPR nanosensor surface is that the MAA monomer used in the polymeric film gives the surface a hydrophilic feature.^50^

**Figure 4:**
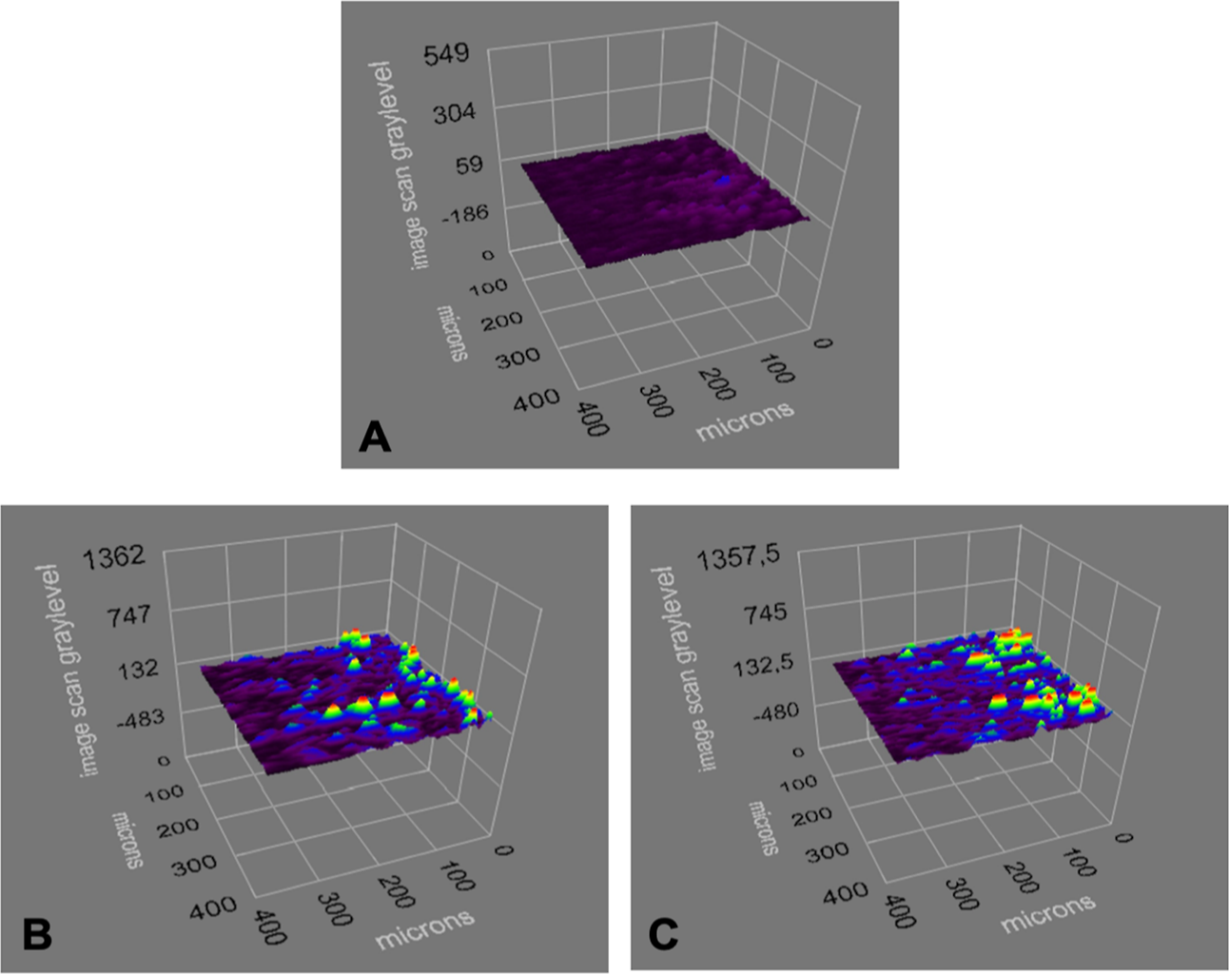
Ellipsometry images: (A) allyl mercaptan-modified SPR chip, (B) adenosine-imprinted SPR nanosensor surface, and (C) non-imprinted SPR nanosensor surface prepared without using the template molecule adenosine.

The polymer thicknesses on SPR nanosensor surfaces were measured by the EP3-Nulling Ellipsometer (Göttingen, Germany). Ellipsometer measurements were made at a wavelength of 532 nm and an incidence angle of 62°. The polymer thicknesses of allyl mercaptan-modified SPR chip surface, adenosine-imprinted, and non-imprinted SPR nanosensor surfaces are 64.4, 119.2, and 115 nm, respectively (Figure 4). It can be seen that the surface thickness is higher on the adenosine-imprinted SPR nanosensor surface. This indicates the presence of molecular cavities on the adenosine-imprinted SPR nanosensor surface compared to the non-imprinted SPR nanosensor surface.

### Kinetic Analysis for Detection of Adenosine

#### Optimization of the MAA/Adenosine Pre-polymerization Complex Ratio

**Figure 5:**
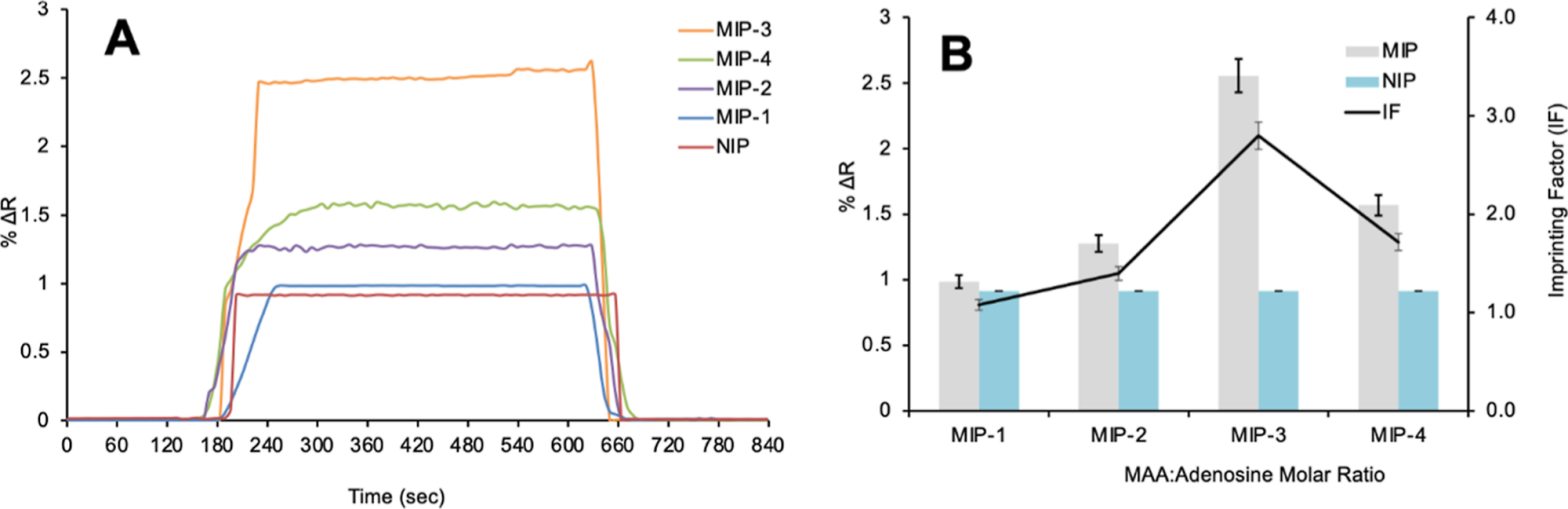
Determination of the imprinting factor using adenosine-imprinted (MIP) and non-imprinted (NIP) SPR nanosensors [(A) sensorgram images of kinetic analyses and (B) column chart representation of the imprinting factor (IF), MAA/Ado 1:1 M with code MIP-1, MAA/Ado 5:1 M with code MIP-2, MAA/Ado 10:1 M with code MIP-3, and MAA/Ado 20:1 M with code MIP-4].

MAA/Ado complexes were prepared in different molar ratios (1:1, 5:1, 10:1, and 20:1 M) to determine the stoichiometric coupling ratios to form the pre-polymerization complex with MAA/adenosine. Adenosine-imprinted (MIP) and non-imprinted (NIP) SPR nanosensors were prepared by keeping the template molecule adenosine ratio constant and varying the amount of monomer MAA. Kinetic analyses were performed with 10 nM adenosine aqueous solution. SPR sensorgrams were monitored in real time, and %Δ*R* values against time were obtained as in Figure 5A. According to the results of the kinetic analysis, the highest imprinting factor was observed in the MIP-3-coded adenosine-imprinted SPR nanosensor prepared with a pre-polymerization complex of 10:1 M (Figure 5B).

#### Determination of Optimum pH Medium

**Figure 6:**
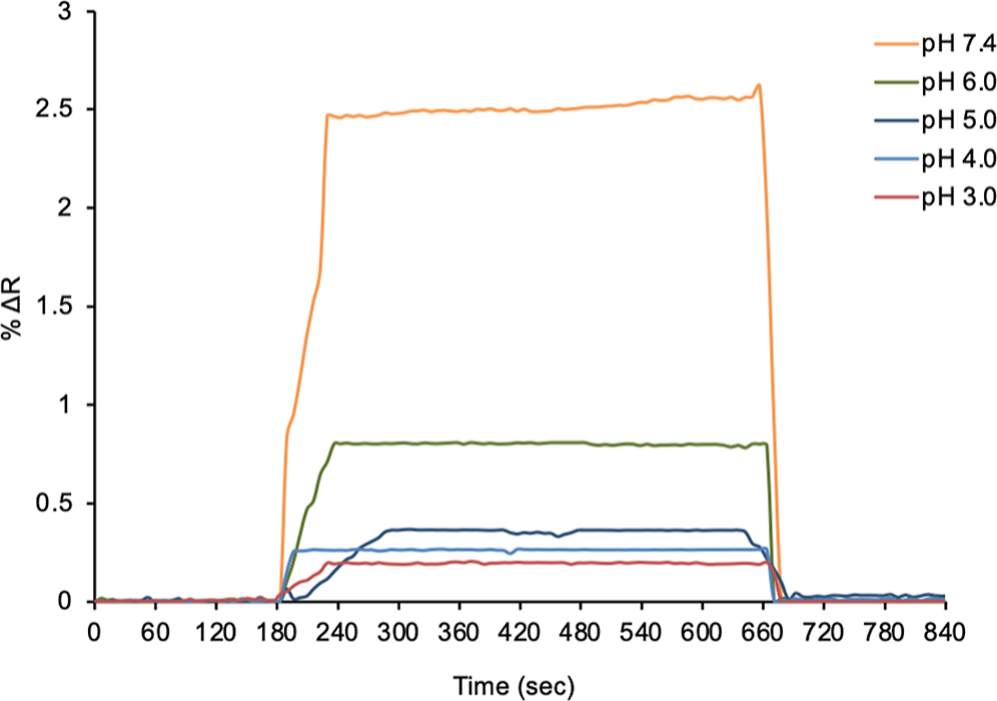
Effective pH range in the adenosine-imprinted SPR nanosensor.

Kinetic analyses were performed with acetate and phosphate buffers at different pHs to determine the ideal pH for the detection of adenosine. The results of kinetic analyses performed with adenosine solutions at 10 nM concentration prepared with acetate pH 3.0, pH 4.0, and pH 5.0 and phosphate buffers pH 6.0 and pH 7.4 are shown in Figure 6. According to the obtained SPR sensorgrams, it was observed in kinetic studies that the highest peak was pH 7.4 phosphate buffer. Due to the decrease in the protonation of the amine group in the adenosine structure, an increase in the SPR signal was observed in the determination of adenosine with increasing pH.^51^ In all kinetic analyses for adenosine detection, adenosine solutions were prepared in pH 7.4 phosphate buffer.

### Real-Time Kinetic Analysis of Adenosine in Aqueous Solutions

**Figure 7:**
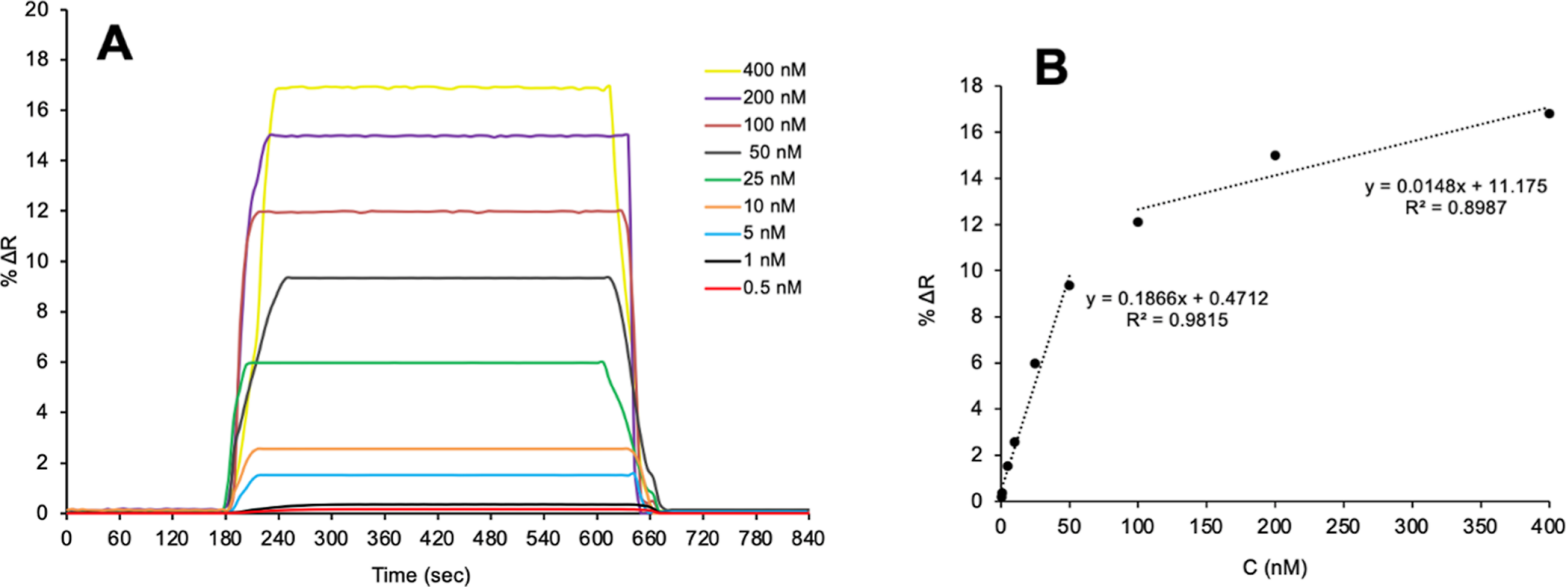
Real-time kinetic analysis (A) and concentration dependency (B) of the adenosine-imprinted SPR nanosensor in aqueous solutions.

For the detection of adenosine, kinetic analyses were performed using solutions prepared at different adenosine concentrations with adenosine-imprinted SPR nanosensors. The obtained sensorgrams from the kinetic analyses with adenosine aqueous solutions prepared at 0.5–400 nM concentrations are given in Figure 7. In kinetic studies, the equilibration buffer pH 7.4 phosphate buffer was passed through the system for 3 min. Then, adenosine solutions prepared at concentrations ranging from 0.5 to 400 nM were passed through the system for 8 min. Finally, 0.1 M NaCl solution was passed through the system for 3 min as the desorption solution. In all kinetic analyses, equilibrium, adsorption, and desorption steps took place in 14 min and were monitored in real time on the SPR system. The % refractive index–time graphs of adenosine solutions applied at different concentrations with adenosine-imprinted SPR nanosensors are shown in Figure 7A.

Figure 7B shows the dependency of adenosine concentrations applied at the adenosine-imprinted SPR nanosensor versus the SPR response signal. The two different binding behaviors of kinetics were evaluated for adenosine because of having two different linear equations. The high regression coefficient for low concentrations of adenosine indicates that binding was achieved with high affinity. The adenosine-imprinted SPR nanosensor is capable of measuring in the 0.5–50 nM concentration range, and the equation *y* = 0.1866*x* + 0.4712 was obtained from the linear graph obtained from the change in refractive index (%Δ*R*) with the increase in concentration with 99% accuracy. In the range of 100–400 nM adenosine concentration, the equation *y* = 0.0148*x* + 11.175 was obtained with approximately 89% accuracy. The refractive index value increased in direct proportion to the adenosine concentration. This shows that as the concentration difference between the adenosine solution and SPR nanosensor surface increases, the driving force between them does as well (Figure 7B).

Two different kinetic analyses were applied for the determination of equilibrium constants for the adenosine-imprinted SPR nanosensor. Association kinetic analysis is an approach based on pseudo-first-order adsorption kinetics. Equilibrium analysis (Scatchard) is used to analyze the data for freely reversible host/guest binding interactions and calculate the total number of binding sites the host has in equilibrium situation.^52^ Kinetic analysis was applied the following equations
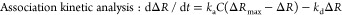
1
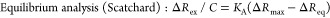
2

The SPR sensor system response is Δ*R*, which measures the signal by binding of adenosine; the concentration of adenosine is *C* as used nM. *K*_A_ (nM^–1^) and *K*_D_ (nM) are the association and dissociation equilibrium constants, respectively, and the association and dissociation kinetic rate constants are *k*_a_ (nM^–1^ s^–1^) and *k*_d_ (s^–1^). The equilibrium, maximum, and experimental subscripts refer to eq, max, and ex, respectively.

**Figure 8:**
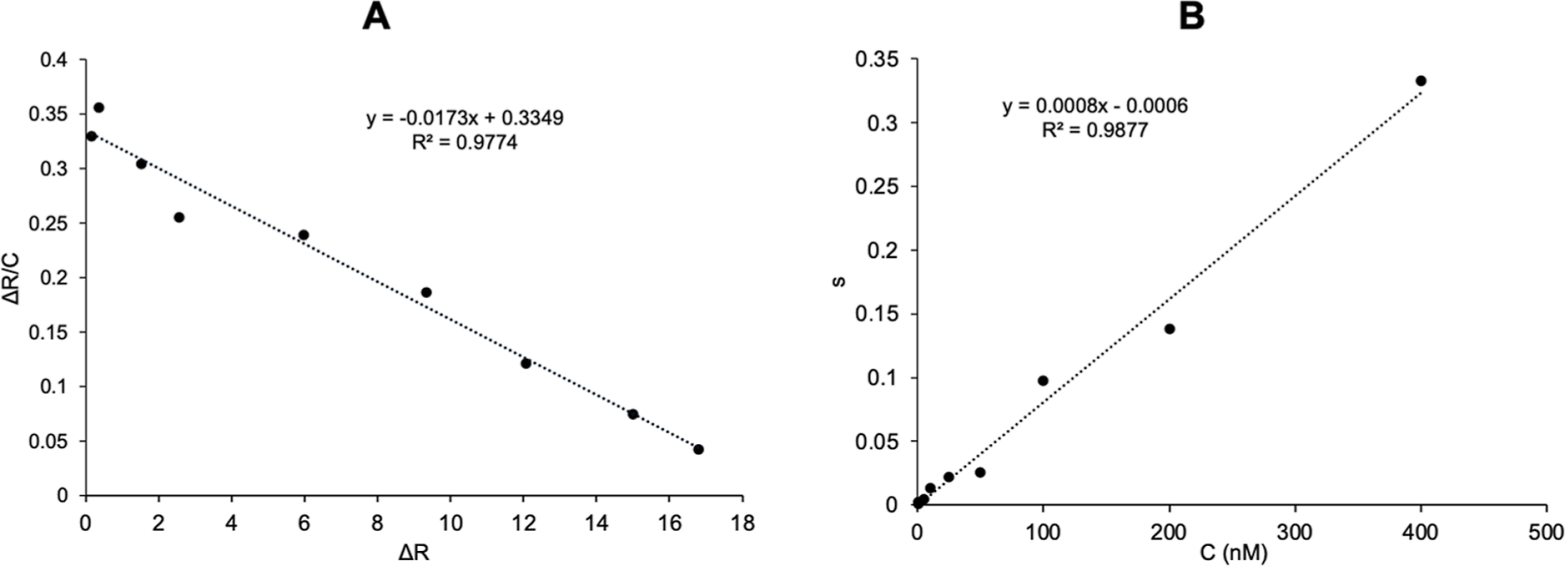
Determination of the kinetic binding constants between adenosine and MIP nanosensor [(A) equilibrium analysis (Scatchard) and (B) association kinetics analysis].

The kinetic binding constants were figured out for association and equilibrium (Scatchard) kinetic analysis. According to these results, it can be seen that the theoretical Δ*R*_max_ value (19.36) calculated in the equilibrium kinetic analysis (Scatchard) is quite close to the experimentally obtained Δ*R*_max_ value (16.8). The coherency between data and model in the terms of correlation coefficient (*R*^2^) showed that association kinetic analysis (*R*^2^: 0.9877) is better fitted than Scatchard analysis (*R*^2^: 0.9774). As can be seen in Table 1, the association kinetic rate constant (*k*_a_: 0.0008 nM^–1^ s^–1^) was higher than the disassociation kinetic rate constant (*k*_d_: 0.0006 s^–1^), and the association equilibrium constant (*K*_A_: 1.133 nM^–1^) was higher than the dissociation equilibrium constant (*K*_D_: 0.75 nM). These data show that adenosine molecules bind to the adenosine-imprinted SPR nanosensor with high affinity (Figure 8).

**Table 1:** Kinetic Parameters of the Adenosine-Imprinted SPR Nanosensor

equilibrium analysis (Scatchard)		association kinetics analysis	
Δ*R*_max_	19.36	*k*_a_, nM^–1^s^–1^	0.0008
*K*_A_, nM^–1^	0.0173	*k*_d_, s^–1^	0.0006
*K*_D_, nM	57.80	*K*_A_, nM^–1^	1.133
*R*(2)	0.9774	*K*_D_, nM	0.75
		*R*^2^	0.9877

The limit of detection (LOD) and limit of quantification (LOQ) values were calculated based on the data obtained in kinetic analysis.
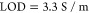
3
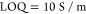
4

In these equations, the *s* value represents the signal value (Δ*R*) received when the equilibrium solution (blind solution) passes over the SPR nanosensor surface, and the *m* value represents the slope formed in the calibration graph.^44^ The Δ*R* value was averaged over 10 measurements. The standard deviation value of the measurements was determined to be 0.0011 for SPR nanosensors. Thus, the LOD was calculated as 0.018 nM, and the LOQ was calculated as 0.061 nM with the equation *y* = 0.1866*x* + 0.4712 of the calibration chart. Several sensor studies in the literature for the detection of adenosine are summarized in Table 2.

**Table 2:** Different Sensor Studies in the Literature for the Detection of Adenosine

used methods	linear range	LOD	refs
electrochemical biosensor	5–2000 nM	5 nM	(53)
electrochemical biosensor	0.37–37.4 μM	0.21 μM	(54)
chemiluminescent biosensor	4 × 10^–13^–1.5 × 10^–14^M	1.04 × 10^–13^M	(55)
fluorometric aptasensor	60–280 nM	21 nM	(56)
colorimetric assay	1.0–5.0 μM	45 nM	(57)
fluorometric aptasensor	10–600 μM	0.30 μM	(58)
SPR nanosensor	0.5–400 nM	0.018 nM	in this study

### Selectivity Analysis

**Figure 9:**
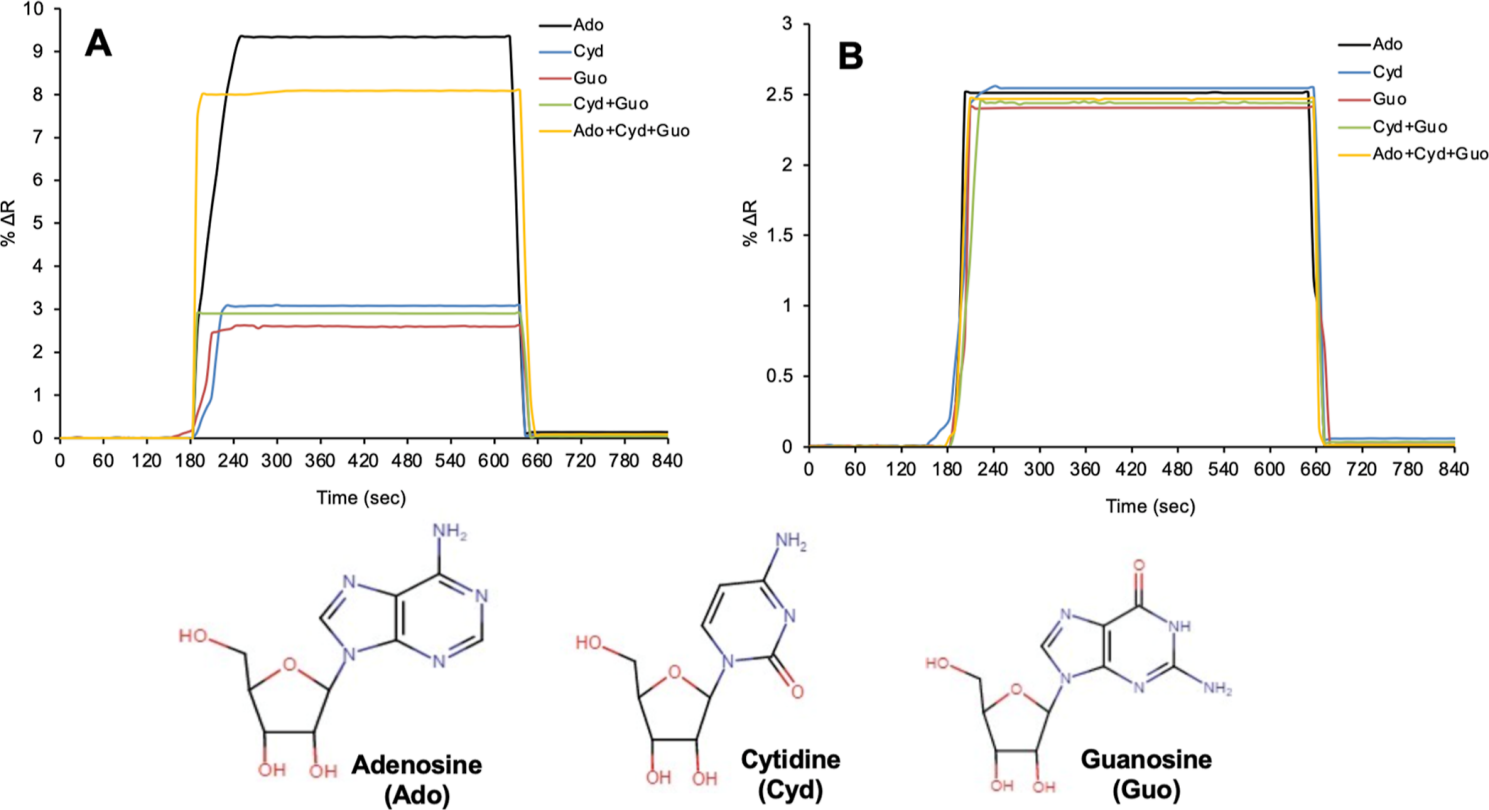
Selectivity studies of adenosine-imprinted (A) and non-imprinted (B) SPR nanosensors.

In the selectivity analysis study, in order to determine the selectivity of the adenosine-imprinted (MIP) and non-imprinted (NIP) SPR nanosensor against adenosine, competitive adsorption studies of Ado, Cyd, Guo, double Cyd + Guo, and triple Ado + Cyd + Guo solutions were performed and kinetic analyses were carried out. When the results were evaluated, the highest refractive index was obtained in the adenosine molecule in the prepared adenosine-imprinted SPR nanosensor (Figure 9A). It was observed that the adenosine-imprinted SPR nanosensor had little specific interaction with guanosine and cytidine molecules and generated a low signal. When the kinetic analysis is performed with the solution formed with cytidine and guanosine nucleosides other than adenosine, it can be seen that the change in the values of the refractive index of the mixture formed with Ado + Cyd + Guo is lower. This indicates that the nucleoside molecules act as a competing agent in the mixture, causing a non-significant reduction in the refractive index change.

The selectivity coefficient (*k*) and relative selectivity coefficients (*k*′) were calculated for both adenosine-imprinted (MIP) and non-imprinted (NIP) nanosensors using the following equations obtained from the kinetic analysis^29^
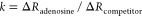
5
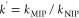
6

When adenosine-imprinted and non-imprinted SPR nanosensors were compared, it was observed that the SPR signal for adenosine molecules decreased from 10.43 to 2.52. The relative selectivity coefficients (*k*′) of the adenosine-imprinted SPR nanosensor for Ado/Cyd and Ado/Guo are 3.836 and 3.427, respectively (Table 3). In addition, the selectivity of the adenosine-imprinted SPR nanosensor over the non-imprinted SPR nanosensor was calculated by the imprinting factor (IF): Δ*R*_MIP_/Δ*R*_NIP_. The imprinting factors of the adenosine-imprinted SPR nanosensor against Ado, Guo, and Cyd molecules were calculated as 4.14, 1.07, and 1.21, respectively. The imprinting factor for adenosine appears to be higher than the IFs of other competing molecules. These results show that the molecularly imprinted polymeric film can significantly increase the adsorption selectivity and that the specific recognition sites are not suitable for other molecules.

**Table 3:** Selectivity and Relative Selectivity Coefficients for Adenosine (Ado), Cytidine (Cyd), and Guanosine (Guo)

	MIP nanosensor		NIP nanosensor		
molecule names	Δ*R*	*k*	Δ*R*	*k*	*k*′
Ado	10.43		2.52		
Cyd	3.08	3.386	2.55	0.988	3.427
Guo	2.59	4.011	2.41	1.046	3.836
Cyd+ Guo	2.90	3.597	2.44	1.033	3.482
Ado +Cyd+ Guo	8.09	1.289	2.47	1.020	1.264

### Detection of Adenosine from Artificial Plasma and Artificial Urine Samples

**Figure 10:**
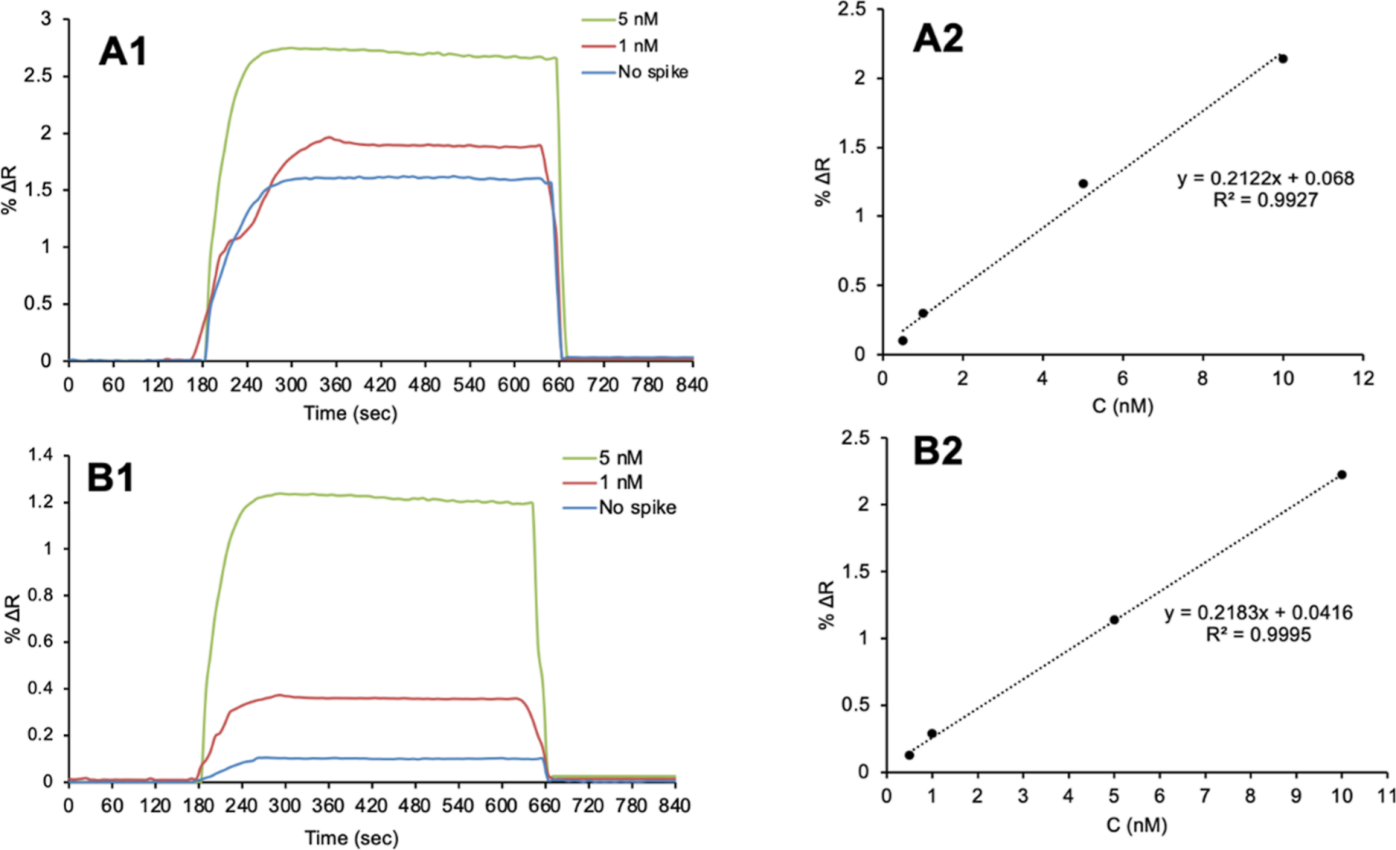
Sensorgrams for adenosine detection from artificial plasma (A1) and artificial urine (B1) and calibration curves for artificial plasma (A2) and artificial urine (B2).

The amount of adenosine from the artificial plasma vs urine samples was determined by kinetic analysis using the adenosine-imprinted SPR nanosensor. First, kinetic analysis was performed by passing artificial plasma to the SPR system. Then, the spiked solutions at 1 and 5 nM concentrations were given to the system, and kinetic analyses were performed. After each kinetic analysis, the adenosine molecules were removed by passing 0.1 M NaCl desorption solution to the SPR system (Figure 10A1).

The amount of adenosine in artificial plasma was calculated from the data obtained from the SPR sensorgrams with %Δ*R* values calculated using the equation *y* = 0.2122*x* + 0.068 (Figure 10B). The accuracy of the data for the detection of adenosine in artificial plasma was found to be *R*^2^: 0.9927. The LOD and LOQ values were also calculated from the kinetic analyses of the adenosine-imprinted SPR nanosensor in artificial plasma samples. The Δ*R* value for the blank solution was determined to be 0.0010 for SPR nanosensors, together with the standard deviation value of the measurements, by averaging the 10 measurements. With the equation *y* = 0.2122*x* + 0.068 of the calibration chart, the LOD and LOQ values were calculated as 0.015 and 0.052 nM in artificial plasma, respectively.

The kinetic analysis steps for the detection of adenosine in artificial plasma were also carried out from the artificial urine solution. Kinetic analyses were performed with adenosine-imprinted SPR nanosensors by adding solutions at 1 and 5 nM adenosine concentrations to the artificial urine solution in Figure 9B1. The LOD and LOQ values were also calculated from the kinetic analyses of the adenosine-imprinted SPR nanosensor in artificial urine samples. The Δ*R* value for the blank solution was determined to be 0.0010 for SPR nanosensors, with the standard deviation value of the measurements, by taking the average of 10 measurements. By using the equation *y* = 0.2183*x* + 0.0416 of the calibration graph, the LOD and LOQ values were determined to be 0.013 nM and 0.046 nM in artificial urine, respectively. Table 4 contains the results of the analysis and comparison of adenosine in artificial plasma and artificial urine samples in the SPR system. To determine the reliability and accuracy of the adenosine-imprinted SPR nanosensor were calculated the recovery (%) for artificial plasma and artificial urine. Approximately 96–98% recovery was obtained for the detection of adenosine in artificial plasma samples.

**Table 4:** Recoveries of Adenosine in Artificial Plasma and Urine Samples (*n*: 3)

	found of Ado (nM)		recovery (%)	
spiked of Ado (nM)	artificial plasma	artificial urine	artificial plasma	artificial urine
1.0 nM	0.988 ± 0.0008	0.982 ± 0.0006	98.8 ± 0.082	96.9 ± 0.058
5.0 nM	4.97 ± 0.0009	4.98 ± 0.0082	99.36 ± 0.019	98.7 ± 0.115

### Examination of Reusability

**Figure 11:**
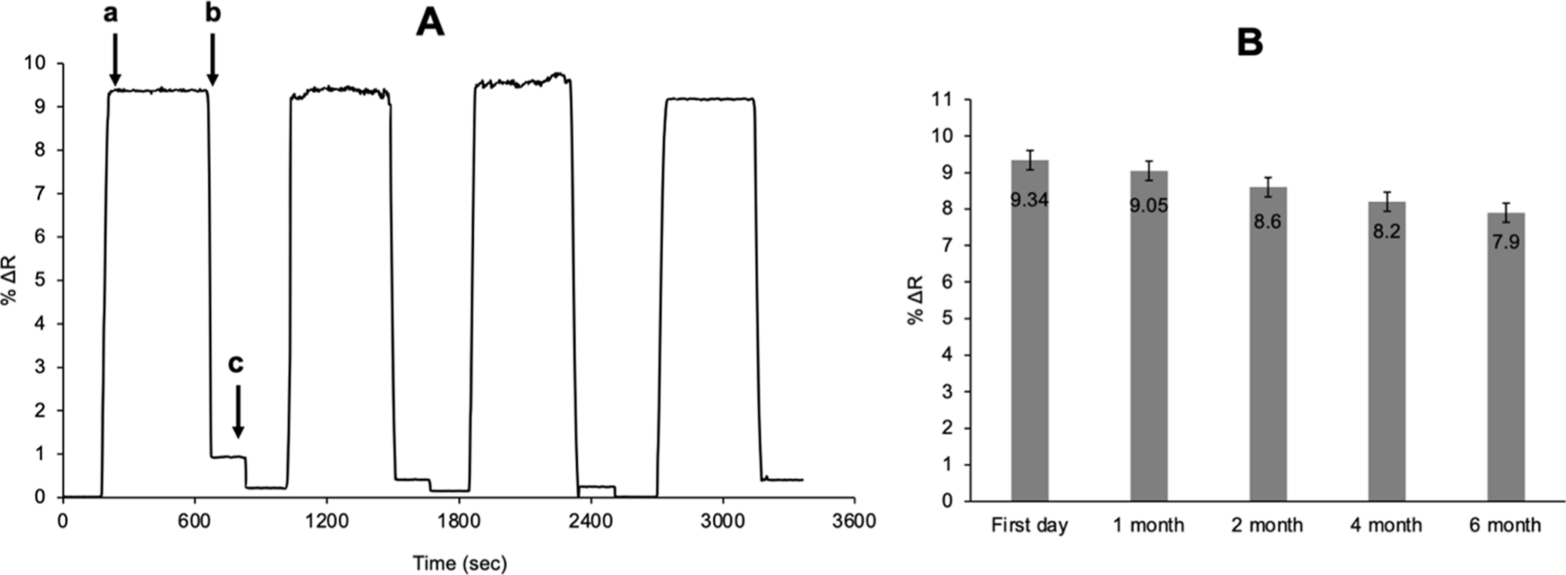
Reusability of adenosine imprinted SPR nanosensors at same day (A) and different day (B) (a: adsorption, b: desorption, and c: regeneration).

The most important advantage in molecularly imprinted SPR-based nanosensors is that the shelf life of the designed nanosensor chip is long and reusable, and it is very advantageous for reuse in the case of using biological molecules as a recognition element. The main reason for this is the deterioration of the three-dimensional structure of the biological molecules in the solutions used, causing rapid degradation, and the change in the nanosensor recognition capacity. It is the decrease in the reproducibility of the designed chip and the solutions used over time due to degradation. By imprinting the structural cavities of the biological material with the MIP method in the prepared SPR nanosensor, its durability and resistance to external conditions increase, and thus, their reusability is ensured for a long time. In order to examine the reusability of the adenosine-imprinted nanosensor, first of all, the pH 7.4 phosphate buffer was passed to ensure the system balance, and then solutions containing adenosine at a concentration of 50 nM were passed through the SPR system five times during the day. After passing each adenosine-containing solution through the system, the 0.1 M NaCl solution was passed to the system for desorption, as shown in Figure 11A. The obtained data after the kinetic analysis were determined with the SPRview software program, and the obtained kinetic analysis results were calculated as %Δ*R* values in the SPR sensorgrams. No decrease in SPR signal was observed during this analysis, which was performed consecutively on adenosine-imprinted SPR nanosensors, and the efficiency value was determined to be 98.06%.

In the presence of 50 nM adenosine, it was observed that there was no significant change in the efficiency of SPR nanosensors when they were reused at different time intervals determined as the first day, first month, second month, fourth month, and sixth month (Figure 11B). It shows that the designed SPR nanosensor was stored at 4 °C for 180 days and maintained its stability as 84.58% when measured again at the end of this period. It has a level of stability resulting from surface immobilization of adenosine on the selective MIP layer on the SPR nanosensor surface. The fact that the stability is protected in this way is due to the fact that the selective voids for adenosine are protected due to polymers by using the MIP technique on the surface.

## Conclusions

Adenosine, which is used as a template molecule in the SPR nanosensor designed in the study, is a nucleoside that is vital for the human body and whose plasma and urine levels are the markers of many diseases. In this study, the adenosine-imprinted SPR nanosensor was prepared by combining the advantages of MIT and the SPR nanosensor for detection of adenosine. The polymeric film imprinting of adenosine on the SPR nanosensor chip surface was achieved by using molecular imprinting technology, thus providing permanent polymeric film formation, which reduces workload and cost and enables precise measurement in nanoscales. According to the analysis results in the studies, the LOD of adenosine in the linear concentration range of 0.1–100 nM in aqueous solutions, artificial plasma, and artificial urine was found to be 0.018, 0.015, and 0.013 nM, respectively. The LOQ values in aqueous solutions, artificial plasma, and in artificial urine were calculated as 0.061, 0.052, and 0.046 nM, respectively. Adenosine was determined from artificial plasma and artificial urine solutions with adenosine-imprinted SPR nanosensors, and approximately 96–98% recoveries were obtained. When the selectivity of adenosine-imprinted SPR nanosensors was examined, the relative selectivity coefficients (*k*′) against the competitor molecules guanosine and cytidine were found to be 3.836 and 3.427, respectively. These values have a much more sensitive value than the results obtained from many adenosine sensor and detection studies in the literature. It is thought that the designed SPR nanosensor will be effective in the medical and research fields in the future.
